# A Brief History of Ferritin, an Ancient and Versatile Protein

**DOI:** 10.3390/ijms26010206

**Published:** 2024-12-29

**Authors:** Paolo Arosio, Gaetano Cairo, Fadi Bou-Abdallah

**Affiliations:** 1 Department of Molecular and Translational Medicine, University of Brescia, 25123 Brescia, Italy; 2 Department of Biomedical Sciences for Health, University of Milan, 20133 Milan, Italy; gaetano.cairo@unimi.it; 3 Department of Chemistry, State University of New York at Potsdam, Potsdam, NY 13676, USA; bouabdf@potsdam.edu

**Keywords:** ferritin, iron metabolism, isoferritins

## Abstract

Ferritin, a highly conserved iron storage protein, is among the earliest proteins that have been purified, named, and characterized due to its unique properties that continue to captivate researchers. Ferritin is composed of 24 subunits that form an almost spherical shell delimiting a cavity where thousands of iron atoms can be stored in a nontoxic ferric form, thereby preventing cytosolic iron from catalyzing oxidative stress. Mitochondrial and extracellular ferritin have also been described and characterized, with the latter being associated with several signaling functions. In addition, serum ferritin serves as a reliable indicator of both iron stores and inflammatory conditions. First identified and purified through crystallization in 1937, ferritin has since drawn significant attention for its critical role in iron metabolism and regulation. Its unique structural features have recently been exploited for many diverse biological and technological applications. To date, more than 40,000 publications have explored this remarkable protein. Here, we present a historical overview, tracing its journey from discovery to current applications and highlighting the evolution of biochemical techniques developed for its structure–function characterization over the past eight decades.

## 1. Introduction

Ferritin is a ubiquitously expressed heteropolymer composed of 24 polypeptide chains assembled into a shell-like structure delimiting a cavity where thousands of iron atoms are stored in a nontoxic ferric form [1]. It was one of the first proteins to be identified and purified. Its stability, distinctive color, and ability to crystallize made it a model for early protein studies. It plays a crucial role in iron metabolism, a biologically essential process for nearly all living organisms, and stands out as a remarkable molecule with a rich history in biochemical research.

Ferritin’s tight regulation by iron levels makes it an attractive subject for gene expression studies, and its polymeric and highly stable structure, which exhibits remarkable protein self-assembly [2], has fascinated researchers for decades. In addition, ferritin stimulates a strong immune response to produce high-affinity antibodies that can be useful for immunological studies. Over the eight decades since its discovery, significant progress has been made in understanding ferritin’s roles in iron oxidation, storage, and release; however, some of the complex biochemical mechanisms underlying these processes remain elusive. A peculiarity of ferritin is the variety of biological functions attributed to this protein over the years [3]. While its primary roles are in iron storage and regulation, it has also been implicated in cellular defense, proliferation, oxidative stress management, and even disease progression. This more than 80-year journey of discovery has paralleled the evolution of biochemical techniques, making ferritin a testament to the growing sophistication of chemical and physical tools used in modern science. The aim of this work is to explore the history of discoveries surrounding this protein, highlighting its remarkable properties and applications that continue to make ferritin a compelling research subject, despite the more than 40,000 published studies. It is thought that history often serves as the foundation for the future, and we hope that this review will inspire and inform future research directions.

## 2. The Origin of Ferritin and Initial Studies

### 2.1. Origin

The history of ferritin began with a discovery by the Czech scientist Vilem Laufberger who in 1937 named “ferritin” an iron-rich compound obtained via the crystallization of horse spleen extract. The groundwork for ferritin discovery was performed by earlier scientists who identified iron deposits in tissues like the liver and spleen. In the late 19th century, scientists such as Perls (1867) discovered iron-containing granules [4], visible as positive Prussian blue stains, in these tissues. These granules, which contained iron oxide and phosphate, varied in color from pale yellow to brown and were particularly abundant in the spleens of horses, accounting for up to 5% of the organ’s dry weight. Further research revealed that these iron deposits also appeared in bone marrow cells following hemoglobin degradation and were later termed hemosiderin. Around the same time, researchers began investigating more diffuse forms of iron in the body. Schmiedeberg in 1894 boiled pig liver homogenates and precipitated the resulting compounds with tartaric acid, isolating a substance containing around 6% iron, which he named “ferratin”. This preparation was initially thought to contain nucleic acids or lipids.

### 2.2. Vilém Laufberger, the Discoverer

Crystallization was one of the few methods available at that time to purify proteins, and was used to identify hemoglobin in 1840, followed by few other proteins that included globulins, albumin, concanavalin A, urease, trypsin and chymotrypsin. Laufberger isolated a highly stable compound containing over 20% iron from horse spleen. His preparation was easily crystallizable using cadmium sulfate and could endure heating at 80 °C, marking a significant discovery [5]. The name “ferritin” was chosen in honor of Schmiedeberg’s earlier work on “ferratin”. These results were confirmed by other groups who identified phosphorus in the ferritin preparation and speculated that it might be a nucleoprotein. Vilém Laufberger, a scientist from Czechoslovakia, initially worked on animal metamorphosis before focusing on insulin purification and ultimately made the groundbreaking discovery of ferritin. His work received some international attention, with presentations in Moscow, Leningrad, and Zurich, leading to a publication in 1937. Later, Laufberger shifted his focus to neurology, publishing a book on the “Theory of Excitement” and pioneering spaciocardiography for heart disease diagnosis. He lived a long and influential life, passing away at the age of 96.

### 2.3. Michaelis and Granick, the Biochemical Bases

Leonor Michaelis, a renowned German scientist celebrated for his work on enzyme kinetics, turned his attention to ferritin later in his career. Although famous for the Michaelis–Menten equation, developed in 1913 with Canadian scientist Maud Leonora Menten, which was foundational for understanding enzyme–substrate interactions, Michaelis faced challenges in establishing a stable career in Germany. He spent three years in Japan before ultimately moving to the United States, where he joined the Rockefeller Institute and focused on oxidation–reduction processes and free radical formation. His interest in ferritin developed during this period, and, in 1942 [6], he demonstrated that ferritin’s iron could be readily removed via reduction with sodium dithionite, introducing the concept of apoferritin, ferritin’s iron-free form. Michaelis encouraged his colleague Sam Granick to study ferritin further, sparking a series of groundbreaking studies from 1942 to 1947.

The research by Granick and Michaelis on ferritin established the foundation for modern iron metabolism studies. Key reviews in 1946 and 1951 [7,8] summarized their findings, highlighting ferritin’s unique ability to crystallize in tissues upon the addition of cadmium sulfate, allowing the visualization and quantification of crystals under a microscope. Their work demonstrated that ferritin could be crystallized from the organs of various species, including horses, humans, dogs, guinea pigs, rats, pigs, cats, and rabbits, though it did not readily crystallize from cows, sheep, deer, fowl, fish, or bullfrogs. They also detected ferritin in tissues like the spleen, liver, bone marrow, kidneys, and testes, where it appeared pale or colorless. A significant finding was the increase in ferritin levels in the duodenum of guinea pigs following oral ferrous sulfate administration, indicating that mucosal ferritin may regulate iron absorption, aligning with the “mucosal block” mechanism described by the Whipple group [9], which involves reduced iron absorption after an oral iron dose. Additionally, purified ferritin enabled antibody production for precipitin reactions that detected ferritin across tissues, though not in the blood, muscle, or the pituitary. Interestingly, horse ferritin antibodies cross-reacted with dog ferritin but not with human apoferritin.

Further research demonstrated that when radioactive iron from hemoglobin or ferric ammonium citrate was injected into animals, it later appeared in liver ferritin, thus showing that liver ferritin stored iron; conversely, when the body’s demand for iron increased, as seen in horses that had undergone extensive bloodletting, ferritin levels in the liver decreased. This indicated that ferritin breakdown occurs in the liver to meet the body’s iron needs, although the exact mechanism remained unclear. Granick and Michaelis also proposed that ferritin might be involved in transporting iron to the fetus via the placenta. Their findings confirmed that ferritin acted primarily as an iron storage molecule, except in the testes, where only apoferritin was found. That extensive work established ferritin’s biochemical and physiological roles, particularly in iron storage and regulation. Their work was followed by that of various groups, notably Tecce in 1952 [10], who first observed ferritin crystals in elasmobranch fishes, broadening ferritin studies beyond mammals.

After making significant contributions to ferritin research, Granick shifted his focus to the biosynthesis of heme and chlorophyll, where he achieved notable recognition.

### 2.4. Mazur and Shorr, the Vasodepressor Material

Following Sam Granick’s pioneering research on ferritin, a new line of inquiry emerged in the late 1940s, led by Shorr and Mazur, who explored ferritin from an entirely different perspective. Their work investigated ferritin’s potential role in regulating blood pressure, particularly its vasodepressor activity.

The research was prompted by observations in the later stages of shock, where capillary dilation causes a significant drop in blood pressure. Shorr and Mazur hypothesized the presence of a “vasodepressor material” (VDM) responsible for this effect in animals undergoing shock. To test this, the authors used the rat mesoappendix to assess the sensitivity of precapillary sphincters to adrenaline (epinephrine). In their assay, they injected the test substance into the rat’s bloodstream and then applied a fixed amount of adrenaline topically to the mesoappendix to measure vasoconstriction [11]. Their experiments revealed that saline washes of the liver and skeletal muscle from animals subjected to shock contained VDM. Notably, anaerobic treatment of the liver was necessary to release VDM. They also discovered that crystalline liver ferritin, but not denatured ferritin, inhibited the epinephrine response in the mesoappendix test. This inhibition was blocked when ferritin was pretreated with antiferritin antibodies, suggesting a role for ferritin in the vasodepressor effect [12]. These findings spurred further research into the chemical properties of ferritin. They found that its vasodepressor activity was linked to the presence of sulfhydryl (SH) groups. Moreover, they observed that cysteine and various forms of iron also exhibited vasodepressor activity, which was similarly inhibited by antiferritin antibodies, raising questions about ferritin’s functions beyond iron storage. Further studies revealed that ferritin could oxidize and deactivate adrenaline in vitro, especially in the presence of hydrogen peroxide [13], providing a partial explanation for its vasodepressor activity and linking ferritin’s ability to release iron with its impact on adrenaline metabolism. Later, the researchers identified products of uric acid metabolism and the enzyme xanthine oxidase as agents that could facilitate iron release from ferritin [14].

Although the theory of ferritin acting as a VDM was not confirmed, the research by Shorr and Mazur made significant contributions to the chemical and physical characterization of ferritin. Importantly, they suggested that ferritin may be present in the circulation and may have physiological roles beyond iron storage. This hypothesis sparked subsequent studies exploring its presence in blood circulation and its various biochemical functions.

### 2.5. Electron Microscopy: Ferritin Identification, Morphology, and Ubiquity

In the mid-1940s, electron microscopy (EM) began to be applied to biological specimens, and, by 1954, Farrant had applied it to study ferritin [15]. This groundbreaking work provided new insights into ferritin’s structure, revealing that its iron core resided within a nearly spherical protein shell approximately 93 Å in diameter. The electron dense iron core, up to 55 Å across, was clearly visible under EM, greatly facilitating the identification of ferritin in tissues. This discovery prompted new research directions, including investigations into ferritin’s relationship with hemosiderin and its potential as a biological tracer.

One notable area of research focused on distinguishing ferritin from hemosiderin, the two main iron storage forms in the body. In 1953, Gabrio, Shoden, and Finch [16] identified differences in the size and morphology of the opaque hemosiderin iron granules. This work was extended by Richter in 1957 [17], who used EM to examine ferritin and hemosiderin in patients with hemosiderosis. Other studies highlighted the identification of ferritin and crystalline lattices within hemosiderin and demonstrated the conversion of colloidal iron injected into animals into both ferritin and hemosiderin. By the mid-1960s, Drysdale and colleagues further advanced the understanding of the interconnected roles of ferritin and hemosiderin in iron storage [18]. Their research demonstrated that ferritin is the primary site for iron accumulation, but its storage capacity is limited. Once tissue iron levels exceed ferritin’s storage limit, excess iron is deposited in hemosiderin. Despite their structural differences, ferritin and hemosiderin were shown to be functionally inseparable, with both releasing stored iron as needed. In fact, ferritin molecules were often found within hemosiderin granules, further highlighting their close association.

The easy recognition of ferritin in EM led to its use as a biological tracer. The iron-rich ferritin from horse spleen, which was relatively simple to purify and easily available, became a valuable tool for studying protein diffusion in tissues and cells, appearing in numerous publications, for example, the Nobel laureate George Palade used it to investigate protein transfer across capillary walls [19]. In addition, ferritin was readily bound to antibodies that were widely used in immunohistochemistry to visualize specific antigens in tissues. This technique, known as immunoelectron microscopy, helped advance the fields of cell biology and molecular medicine, allowing scientists to label and track various biomolecules in precise locations within cells. In this way, EM played a crucial role in studying ferritin’s structure and tracing its interactions in the body, with numerous applications in cell biology and immunology.

EM also enabled researchers to identify ferritin iron cores in various organisms beyond mammals. These studies showed that ferritin is present across the biological world. It was detected in the eggs and embryos of *Rana pipiens* [20]; in various mollusks [21], including *Corbicula sandai* [22]; in the dental cells of urodeles [23]; in snails [24]; in fungi [25]; in octopus and tuna fish [26]; and in the plasma of birds [27]. Further research revealed the presence of ferritin in plants, where it was referred to as “phytoferritin.” Unlike in animals, where ferritin is found in the cytosol, plant ferritin localizes in the plastids, a type of organelle [28]. Ferritin was also detected in plant roots [29] and in the root tips of beans [30]. It was found in various other plants, including the leaves of *Xanthium* [31]. Craig and William explored the connection between phytoferritin and viral infections, suggesting a potential role in plant immune responses [32]. This series of EM-based studies confirmed the widespread presence of ferritin across various species, highlighting its fundamental role in essential biological processes such as iron storage, detoxification, and regulation across all eukaryotic life forms.

## 3. The 1960s and the Novel Biochemical Techniques

### 3.1. Early Studies of Ferritin Biosynthesis and Structure

Sam Granick’s pioneering work revealed that ferritin accumulation is rapidly induced by iron in animals, making ferritin an excellent model for studying the regulation of gene expression and protein synthesis. As a result, various laboratories began investigating ferritin biosynthesis. In 1955, Fineberg and Greenberg [33] conducted research on guinea pigs, showing that ferritin biosynthesis is accelerated by iron, with apoferritin (the protein shell without iron) being produced before ferritin [34]. Later, Richter explored the ferritin biosynthesis in liver cells and HeLa cells [35], followed by studies from Shoden and Sturgeon [36] and Friedberg et al. in guinea pigs [37]. A significant breakthrough occurred in 1965 when Drysdale and Munro demonstrated that actinomycin D, a transcription inhibitor, failed to inhibit iron-induced ferritin synthesis in rats, proving that ferritin synthesis is mainly regulated at the post-transcriptional level [38].

In 1957, Loewus and Fineberg [39] discovered the conditions for incorporating iron into apoferritin, thereby reconstituting ferritin in vitro. During the 1960s, the development of new biochemical and biophysical techniques facilitated a deeper understanding of ferritin’s structure and function. X-ray diffraction studies by Kleinwachter [40] and Harrison [41] provided insights into ferritin’s symmetry, size, and shape. Concurrently, Crichton and colleagues focused on the biochemical characterization of ferritin, employing proteolytic enzymes, circular dichroism, and other techniques [42]. Radola et al. applied gel filtration analysis for molecular weight determination [43], while starch electrophoresis revealed that liver ferritin could be separated into two bands, potentially representing ferritin monomers and oligomers [44]. The introduction of polyacrylamide gel electrophoresis (PAGE), first described in 1959 by Raymond and Weintraub [45], offered greater resolution in protein separation. In 1965, its use by Richter revealed the differences in the ferritins from normal versus neoplastic cells [46]. Subsequent studies confirmed that the ferritin from different tissues or species exhibits variations in electrophoretic mobility, suggesting the potential existence of tissue-specific forms of ferritin. Initially, these electrophoretic techniques were cumbersome, as the runs were conducted in tubes, making band alignment very challenging. This issue was resolved with the development of slab gel electrophoresis, which greatly improved band visualization. Another significant advancement was the introduction of isoelectric focusing in 1971 by Urishizaki et al. [47]. This technique demonstrated that even crystallized horse spleen ferritin could be separated into several bands with different electric charges. When applied to human specimens, the most acidic ferritin bands were found in tumor tissues, giving rise to the concept of carcino-fetal isoferritins [48]. This finding sparked further interest due to its parallel with the recently discovered carcinoembryonic antigen (CEA) and alpha-fetoprotein.

A major advancement in electrophoresis came with the development of SDS-PAGE in 1970 by Laemmli [49], which allowed for the analysis of denatured proteins and their subunits. This technique was used to determine whether ferritin consisted of one or multiple subunit types, initially yielding conflicting results. However, it was eventually demonstrated that human ferritin is composed of two distinct subunit types: H (heavy, heart, MW 21,000) and L (light, liver, MW 19,000), with the H subunit being more prevalent in the heart and the L subunit in the liver. These subunits were later found to have different amino acid compositions and to be encoded by separate mRNAs. This clarified that the isoferritins observed in different tissues were due to the varying proportions of the H and L subunits, with the more acidic ferritins being richer in the H subunit [50]. These discoveries marked a significant leap in understanding ferritin’s complex structure, regulation, and functional diversity across different tissues and species.

### 3.2. Radioimmunoassays and Serum Ferritin

The studies of Mazur and Shorr suggested that ferritin might circulate in the blood and have additional functions. Some research groups used antibodies to detect ferritin in the blood, but the low sensitivity of the techniques available at the time only allowed its detection in the serum of animals experiencing experimental shock [51] but not in healthy individuals. It was not until the development of sensitive radioimmunoassays that ferritin was reliably detected in the serum of all individuals, including healthy subjects. In 1972, Addison et al. demonstrated that serum ferritin concentrations were higher in individuals with iron overload and lower in those with iron deficiency, establishing ferritin as a useful marker for assessing body iron status [52]. This discovery led to the widespread use of serum ferritin measurements as a clinically important diagnostic tool for anemia, iron-related diseases and siderosis [53]. Initially, competitive assays using radiolabeled human ferritin and antibodies for horse spleen ferritin were employed. Later, the symmetrical structure of ferritin made it an ideal model for developing more advanced two-site (sandwich) immunoassays. These sensitive assays also revealed that ferritin is present in various body fluids, including cerebrospinal fluid (CSF), urine, milk, and colostrum, although extracellular ferritin amounts are much lower than intracellular concentrations.

While serum ferritin became—and continues to be—a key clinical indicator for diagnosing iron deficiency (or overload) and other diseases, only a limited number of studies have focused on its specific characteristics, showing that it is iron-free or iron-poor and shares structural similarities with liver apoferritin [54]. It was found to predominantly consist of the L subunit, which is partially glycosylated and recognized by the lectin concanavalin A [55,56]. However, open questions remained about its origin and the mechanisms through which it enters the bloodstream. Konijn et al. showed that ferritin synthesis occurs on both free and membrane-bound polyribosomes, suggesting the possibility of active secretion [57]. Moreover, the presence of ferritin mRNAs on bound polysomes was found to be elevated in rat livers during experimental inflammation [58] and in experimental tumors [59]. However, ferritin subunits lack canonical leader peptides and in vitro translation experiments did not reveal the post-translational modifications typical of secreted proteins [60] and ferritin mRNA injection into Xenopus oocytes did not result in ferritin secretion in the medium [59].

The discovery that ferritin exists in extracellular spaces spurred investigations into potential non-iron-related functions. Matzner et al. [61] found that ferritin had a suppressive effect on lymphocyte function, indicating it might have immunomodulatory roles, in addition to its well-known function as an iron storage protein. The dual roles of ferritin, both as an intracellular iron storage protein and a potential extracellular signaling molecule, continue to intrigue researchers, with many studies focused on unraveling the complexities of its secretion, circulation, and broader physiological functions. Building on the groundbreaking work on monoclonal antibodies by Köhler and Milstein of 1975 [62], the production and characterization of the first monoclonal antibodies for heart ferritin [63] marked a significant advancement. Subsequently, monoclonal antibodies targeting both ferritin subunits (H and L) were developed [64], offering valuable tools for further ferritin studies.

### 3.3. Ferritin Crystallographic Structure

**Figure 1:**
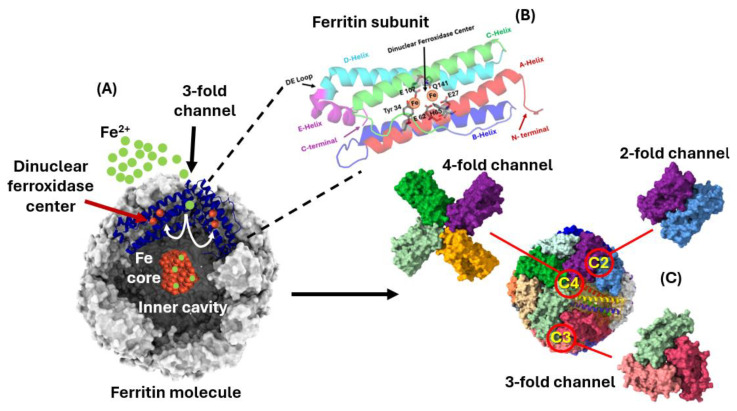
(**A**) Overall structure of ferritin depicting iron access to the ferroxidase center through the 3-fold channel and an iron core inside the ferritin cavity, (**B**) the helical structure of one subunit, and (**C**) the ferritin subunit interfaces at the C2 (dimer), C3 (trimer), and C4 (tetramer) symmetry. The figure is a modified version from Bou-Abdallah et al. [66].

A major milestone in ferritin research was the publication of the crystallographic structure of horse spleen ferritin in 1984 by Ford et al., which revealed that its subunits are composed of a bundle of four alpha helices along with a fifth helix at the C-terminus positioned at an acute angle to the bundle (Figure 1). The 24 subunits self-assemble into an almost spherical molecule exhibiting two-, three-, and four-fold symmetries [65] with a large cavity approximately 8 nm in diameter where iron is stored as ferrihydrite granules containing up to 4000 atoms. The overall structure of this 500 kDa protein includes pores along the three- and four-fold symmetry axes, which were proposed as potential routes for iron entry and exit. The resolution of this structure was complex and painstaking, involving the use of heavy atom derivatives, but it greatly simplified the subsequent determination of ferritin structures from many different origins. Moreover, it was critical for understanding the mechanism of iron incorporation into the protein cavity, which was suggested to occur through the hydrophilic three-fold channels rather than the hydrophobic four-fold channels.

## 4. The 1980s and the Role of Molecular Biology

### 4.1. Cloning the Ferritin Genes

The 1980s witnessed rapid advancements in molecular biology, promoted by the first publication of Molecular Cloning: A Laboratory Manual by Sambrook et al. [67]. This influential work became an essential resource for molecular biology research, supporting numerous laboratories in the identification and cloning of genes. The cDNAs for the ferritin chains were cloned by various groups: initially, in 1983, the cDNA for the rat L subunit by Brown et al. [68], followed by the human L subunit cDNA by Costanzo et al. [69]; shortly thereafter, the cDNA for the H chain was cloned by Boyd et al. [70]. However, identifying the functional ferritin genes was complicated by the discovery of multiple bands in Southern blots, which were later attributed to inactive retro-pseudogenes [71]. The functional human L-ferritin gene was mapped in 1983 to chromosome 19 by Caskey et al. [72], and the H-chain gene was localized to chromosome 11 by Worwood et al. [73]. Subsequently in 1987, the cDNAs for both the human H and L chains were cloned into prokaryotic expression vectors, and the corresponding ferritins were expressed in *Escherichia coli* as recombinant proteins [74,75]. This work demonstrated that the H chain possesses ferroxidase activity, which was absent in the L chain [76]. Recombinant H-ferritin was then crystallized for 3D structural determination, identifying the ferroxidase site within the four-helix bundles of the subunit, composed of two iron-binding sites [77]. Site-directed mutagenesis was employed on recombinant ferritins to explore the residues involved in iron uptake and the stability of the protein. The ferroxidase site was identified as essential for iron metabolism [78]. This breakthrough paved the way for further research into ferritin’s function, particularly the detailed mechanisms of iron oxidation and storage within the ferritin molecule.

### 4.2. Regulation of Ferritin Expression, the IRE/IRP Machinery

Cloning ferritin cDNA was also fundamental for investigating the control of ferritin expression. In particular, significant advances were made in the area of iron-dependent post-transcriptional regulation cited above. Early studies by Aziz and Munro revealed that both ferritin subunits (H and L) are similarly stimulated in response to increased iron levels in mammals [79]. Confirming the remarkable foresight of the post-transcriptional model proposed by Munro and colleagues [80], this regulation was traced to a conserved sequence located in the 5′ untranslated region (UTR) of the ferritin mRNA, termed the iron-responsive element (IRE) [81]. The discovery of the IRE was crucial in understanding how cells regulate ferritin synthesis in response to iron availability. The proteins responsible for binding to the IRE and regulating ferritin translation, known as iron regulatory proteins (IRP1 and IRP2), were identified the following year. This discovery was made by comparing the translation efficiency of mammalian (reticulocyte lysate) and plant (wheat germ extract not containing IRPs) in vitro systems [82].

**Figure 2:**
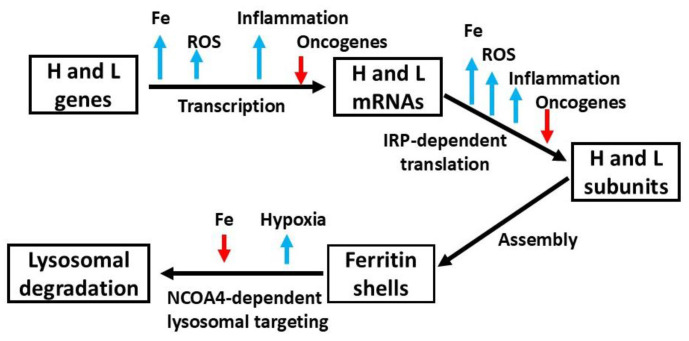
Schematic diagram showing the regulatory mechanisms of ferritin expression. Fe indicates both Fe (II) and Fe (III), which are in equilibrium inside the cell, with a prevalence of Fe (II) inside the cell. The blue arrows pointing upward indicate stimulation, while the red ones pointing downward indicate repression.

In the same year, a similar binding activity was documented in rat liver [83]. Importantly, it was shown that the IRP binding activity could be measured with the electrophoretic mobility shift assay (EMSA) using radioactive IRE probes, a highly sensitive method that was employed in many studies on cellular iron homeostasis. The IRPs regulate ferritin production by binding to the IRE of both H and L mRNAs under low-iron conditions, blocking translation and thus reducing ferritin synthesis. Conversely, when iron levels are sufficient, IRP1 assembles an iron sulfur cluster, becoming inactive, while IRP2 undergoes degradation, allowing ferritin synthesis to proceed (Figure 2). This regulatory mechanism not only clarified how ferritin expression is finely tuned in response to iron availability but also underscored the broader importance of post-transcriptional regulation in controlling protein expression [84]. As IREs were later discovered in the mRNAs of other key proteins of iron metabolism (see below), these findings paved the way for further exploration of iron metabolism and related disorders.

### 4.3. The Importance of the IRE/IRP Machinery

The IRE/IRP machinery became a fascinating example of post-transcriptional regulation, particularly after it was discovered that this regulatory system extends beyond ferritins and iron storage. It also controls other proteins involved in iron uptake (transferrin receptor, TfR1), export (ferroportin), and utilization (erythroid 5-aminolevulinic acid synthase (eALAS, the key enzyme in heme synthesis) [85]. Gray and Hentze [86] first highlighted this broader regulatory role, which extends to other proteins related to iron metabolism indicated above. Detailed studies showed that IRP1 inhibits the binding of eIF4F to the 5′ untranslated region of ferritin transcripts, thereby blocking translation initiation [87]. Interest in the IRE/IRP system grew significantly with the discovery of its involvement in a genetic disorder called hereditary hyperferritinemia cataract syndrome (HHCS). This autosomal-dominant condition results from mutations in the IRE of the L-ferritin mRNA, which diminish the binding affinity of IRPs, leading to the unregulated overproduction of L-ferritin. Elevated serum ferritin levels in these patients are accompanied by early-onset bilateral cataracts. The genetic basis of HHCS was elucidated almost simultaneously in 1995 by two groups in France and Italy [88,89]. The close relationship between HHCS-dependent hyperferritinemia and the L-subunit ferritin content in mononuclear blood cells strongly suggested that the latter cells are the source of serum ferritin [90], a hypothesis that was confirmed many years later (more below).

### 4.4. Novel Ferritin Functions

Once the fundamental aspects of ferritin were understood and the proper experimental tools became available, research on ferritin diversified into several important directions. One of the notable findings came from Konijn et al., who demonstrated that inflammation affects ferritin synthesis (Figure 2), suggesting a link between ferritin and the body’s response to inflammatory processes [91]. In 1980, Dorner et al. published a key study showing that ferritin synthesis occurs in T lymphocytes [92], expanding the understanding of ferritin’s role in immune function. A particularly important discovery was made by Broxmeyer, who found that acidic ferritins exhibit an inhibitory effect on granulocyte-macrophage progenitor cells [93]. The same group later showed that the myelosuppressive activity of ferritin was related to the H chain’s ferroxidase activity [94]. These findings highlighted a functional connection between ferritin’s role in iron metabolism and immune regulation, especially in hematopoietic processes. Other important discoveries during this period included the observation by Konijn et al. that ferritin is taken up by erythroid precursor cells for heme synthesis [95], underscoring ferritin’s contribution to iron utilization in red blood cell development. Furthermore, Pollack and Campana discovered ferritin receptors on immature red blood cells [96], contributing to the understanding of how ferritin mediates iron transport and uptake in developing erythroid cells. Moreover, in 1989, Fracanzani et al. made a key observation regarding hemochromatosis, finding a lack of ferritin in the duodenal absorptive epithelial cells of patients [97], which was likely due to increased IRP binding activity [98]. This was in line with the previously suggested role of ferritin [3,5] in the hypothesis of a “mucosal block” in iron absorption regulation. Collectively, these studies expanded the scope of ferritin research, establishing its role beyond simple iron storage and its involvement in immune regulation, cancer treatment, and iron homeostasis in various diseases (Table 1). Additionally, studies by Leichner et al. using radiolabeled anti-ferritin antibodies to target primary liver cancer [99] were followed by various other tumor-targeting studies using radio-iodinated antihepatocellular carcinoma ferritin [100]. These efforts highlighted the potential of ferritin-based therapies for cancer diagnosis and treatment.

### 4.5. Ferritins in Animals, Plants, and Bacteria

Although the primary focus of research remained on human and mammalian ferritins, several laboratories explored ferritins from other species and phyla, providing valuable comparative insights. For example, Bottke [101] discovered a secreted ferritin in snails, marking an early observation of ferritin functioning beyond intracellular iron storage. In plants, ferritin was characterized by the presence of an N-terminal transit peptide, a feature enabling its transport into plastids [102], emphasizing a specialized role for plant ferritins in different cellular compartments. Bacterial ferritins also became a major area of interest. Research identified three distinct types of bacterial ferritins. One type, structurally similar to animal ferritins (FTN) was first identified in *E. coli* [103]. Another type, termed bacterioferritin (BFR), was found to contain 24 heme groups, with different iron storage and oxidative properties [104]. A third type, initially identified in *Listeria innocua* by Bozzi et al. [105], was composed of 12 subunits and was previously referred to as DPS (DNA-binding proteins from starved cells). This ferritin variant garnered attention because its crystallographic structure revealed that its subunits also formed four-helical bundles, resembling those found in other ferritins [106]. The DPS ferritin was shown to possess ferroxidase activity, with the active site localized at the interface between the subunits [107]. Further studies on ferritins from various organisms, including *E. coli*, *Azotobacter* and pea seeds [108], contributed to a broader understanding of the structural diversity and functional specialization of ferritins across different species. These studies highlighted the evolutionary conservation of ferritin’s core structure while revealing distinct adaptations in specific species, thus enriching the overall knowledge of iron metabolism across biological systems.

**Table 1:** Some functional roles attributed to ferritin.

Function	Experimental Model	Reference
Iron storage	Guinea pigs and other animals	Granick, 1951 [7]
Vasodepressor material	Rat	Mazur et al., 1950 [109]
Antidiuretic	Rat	Shorr et al., 1950 [110]
Serum ferritin, marker of iron status	Humans and other mammals	Addison et al., 1972 [52]
Inhibitor of granulocytes-macrophage progenitors	Cultured human cells	Broxmeyer et al., 1982 [93]
Radiolabeled antiferritin antibody targets liver cancer	Human patients	Leichner et al., 1984 [99]
Source of iron for ROS formation	Cell-free systems	Reif, 1992 [111]
Cytoprotective antioxidant	Endothelial cells	Balla et al., 1992 [112]
Delivery of iron to erythroid precursor cells	Cultured erythroid cells	Konijn et al., 1994 [95]
Regulated in oncogenesis	Transfected HeLa and B cells	Bevilacqua et al., 1997 [113] Wu et al., 1999 [114]
Binds kininogen	Human serum	Torti et al., 1998 [115]
Binds microtubules	Mouse neuroblastoma cells	Hasan et al., 2006 [116]
Inhibitor of calcification and osteogenesis	Aortic smooth muscle cells	Zarjou et al., 2010 [117]
Stabilizes HIF1α	Dendritic cells	Siegert et al., 2015 [118]
Regulates ferroptosis	Pulmonary fibroblasts	Park and Chung, 2019 [119]
Stimulates inflammasome	Rat hepatic stellate cells	Fernandez-Rojo et al., 2024 [120]

### 4.6. Recombinant Ferritins: Stability and Function

Human ferritin-H was among the first multimeric proteins to be expressed recombinantly in *E. coli*. It was produced in a soluble and stable form with good yields and was subsequently modified through site-directed mutagenesis to investigate its structural stability and its interaction with iron. Alterations in the two-fold symmetry axis inhibited ferritin assembly, while changes in the three- and four-fold axes mainly reduced stability without entirely preventing function [121]. Crucially, it was shown that iron enters and exits through the hydrophilic three-fold channels, while the four-fold channels are thought to be permeable only to protons. Efficient and rapid iron incorporation requires both the ferroxidase activity of the H chain and the nucleation center of the L chain inside the ferritin cavity [122]. Research also demonstrated the formation of a transient blue Fe (III)-tyrosinate complex during the early steps of ferroxidase activity, as reported by Waldo et al. [123]. Efforts to introduce a functional ferroxidase center into the L chain were initially hampered by mutations that disrupted subunit folding. However, this issue was resolved by co-assembling mutant L chains with wild-type L chains, yielding functional molecules with ferroxidase activity [124]. Takagi et al. [125] further suggested that the three-fold channel could undergo partial unfolding under mild conditions, facilitating iron exchange between the ferritin core and its external environment.

A significant breakthrough occurred with the successful modification of the iron core chemistry, transforming it from ferrihydrite into iron sulfide. This advancement paved the way for the use of ferritin as a cargo carrier for various mineral compounds. This was exemplified by a bioinorganic composite created within ferritin by Douglas et al. [126], highlighting ferritin’s potential in nanotechnology and materials science.

In addition to these structural insights, a key milestone enabled by the availability of recombinant ferritins was the establishment of an international standard for serum ferritin based on recombinant human L-ferritin [127]. This marked the first standardized measurement of serum ferritin levels, with significant clinical implications for diagnosing iron-related disorders.

### 4.7. Other Ferritin Functions

Several additional studies have expanded our understanding of ferritin’s functions (Table 1). Cai et al. reported the presence of ferritin in the nuclei of mammalian cells, specifically in corneal epithelial cells [128], while Pountney et al. found nuclear ferritin in human K562 cells, suggesting new roles for ferritin beyond iron storage [129]. Torti and Torti discovered that ferritin could bind to kininogen, a multifunctional protein [115], while Hulet et al. identified ferritin binding sites in the mouse brain [130], which might be involved in iron transfer to brain cells. Notably, further studies on ferritin regulation revealed an association between ferritin and oncogenic pathways; in fact, the gene product of E1A, a viral oncogene [113], and the proto-oncogene c-MYC [114] were found to repress ferritin transcription (Figure 2). Another discovery was that ferritin forms dynamic oligomers that associate with microtubules, offering insights into its structural interactions within cells [116]. These findings indicated novel regulatory and functional roles for ferritins in cellular processes beyond simple iron storage, broadening the scope of research in this area.

### 4.8. Ferritin and Oxidative Stress

During this period, several intriguing findings expanded the understanding of ferritin beyond its traditional role in iron storage. While ferritin may act as a chemical source of iron contributing to oxidative damage [111], H-ferritin was shown to reduce lipid peroxidation in vitro [131] and to function as a cytoprotective antioxidant, particularly in endothelial cells by mitigating oxidative stress, according to Balla et al. [112]. Oxidative stress was shown to induce the coordinated transcriptional and translational regulation of ferritin, as initially demonstrated in vivo by Cairo et al. [132] and later confirmed by Tsuji et al. [133], who precisely characterized an antioxidant response element in the regulatory region of the H subunit, highlighting the protein’s role in the cellular response to stress (Figure 2).

## 5. The New Century

### 5.1. KO Mice, Mitochondrial Ferritin, and Neuroferritinopathy

The early 2000s brought significant advancements in the understanding of ferritin’s functions also due to the development of a knockout (KO) mouse model lacking the H-ferritin subunit. A study by Ferreira et al. [134] revealed that H-ferritin deletion results in embryonic lethality, highlighting the essential role of ferritin’s ferroxidase activity in development and reinforcing the idea that ferritin is crucial in most living organisms. A second landmark discovery of 2001 was the identification of neuroferritinopathy, a rare genetic disorder first described in England that affects the L-ferritin subunit [135]. This neurological disorder is a form of late-onset parkinsonism with dominant transmission. It is caused by nucleotide insertions or duplications at the 3′ end of the L-ferritin coding sequence that alter the C terminus of the protein and lead to the accumulation of iron precipitates in tissues, especially in the brain. A third major milestone of 2001 was the discovery of mitochondrial ferritin (MtF) by Levi et al. [136], which is encoded by an intronless gene. The gene product contains a long N-terminal sequence for mitochondrial targeting, which is cleaved to form a mature protein similar in size and function to the H-ferritin subunit, as it is endowed with ferroxidase activity. This discovery was considered important because mitochondria, being involved in both heme synthesis and Fe-S cluster formation, are critical for intracellular iron processing and are a major source of reactive oxygen species (ROS). MtF gained further attention because of its overexpression in the red blood cells of patients with sideroblastic anemia [137]. A more detailed analysis in mice showed its presence primarily in cells with high metabolic activity, such as those in the heart, spermatozoa, and kidneys, but not in other cells [138]. Interestingly, knockout mice lacking MtF did not display obvious phenotypes, except for increased sensitivity to the cardiotoxic effects of doxorubicin, a chemotherapeutic drug that mainly targets the mitochondria, and reduced male fertility [139]. This suggests a specific role for mitochondrial ferritin in maintaining cellular homeostasis in energy-intensive tissues. MtF was later found in plants and Drosophila [140,141].

### 5.2. Ferritin Receptors

Given the existence of circulating ferritin and its role in hematopoiesis, as demonstrated by Broxmeyer’s studies, the search for ferritin receptors also progressed. Various earlier reports suggested the presence of ferritin binding sites on the cell membrane of mammalian cells, but their precise identity remained elusive. In 2005, Chen et al. identified TIM-2 [142] as a receptor for H-ferritin endocytosis in mouse B cells and in the liver and kidney. However, TIM-2 lacks a human homolog, leaving the human H-ferritin receptor unknown until Li et al. [143] discovered that TfR1 binds and internalizes H-ferritin in human cells. Further progress in 2014 identified SCARA5 as the receptor for L-ferritin, with potential implications in retinopathy [144].

### 5.3. Novel Ferritin Structures

**Figure 3:**
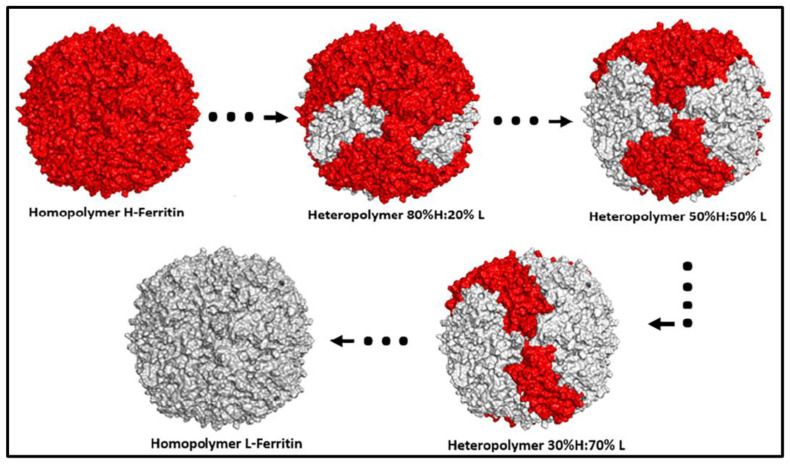
Computer-generated schematic of homopolymer H- and L-ferritin and heteropolymer ferritin with different H and L ratios.

In parallel, structural studies continued to shed light on ferritin’s diversity. One significant finding was the serendipitous crystallization in 2005 of a secreted ferritin from an insect species, which was found to be a heteropolymer composed of two distinct subunit types forming heterodimers. This marked the first 3D structure of a ferritin heteropolymer, achieved by Hamburger et al. [145]. Additional crystallographic studies unveiled the structure of tetrahedral open-pore ferritin from the hyperthermophilic archaeon *Archaeoglobus fulgidus* [146]. Another milestone was the structural elucidation of IRP1 complexed with the IRE motif in ferritin mRNA [147], providing deeper insights into the post-transcriptional regulation of iron metabolism. More recently, a system was engineered that permitted the synthesis of human heteropolymeric ferritins with different H- to L-subunit ratios [148], thus allowing the study of the 3D structures of the isoferritins with different H- and L-chain compositions (Figure 3) [66].

Overall, these discoveries helped connect ferritin’s functions to a broader array of biological processes and highlighted its importance in cellular homeostasis, the oxidative stress response, and iron regulation across different organisms and tissues.

### 5.4. Iron Trafficking to and from Ferritin, Chaperones, and Ferritinophagy

The process of iron incorporation by ferritin has been the subject of extensive research, with early studies showing that ferritin readily takes up iron in vitro. However, the in vivo mechanism remained elusive until, in 2008, an ingenious system in yeast was used to identify a cytosolic iron chaperone named PCBP1 that delivers iron to ferritin and other iron-dependent proteins [149]. This discovery revealed a regulated and controlled system of delivering iron to ferritin, challenging earlier assumptions of passive iron incorporation.

**Figure 4:**
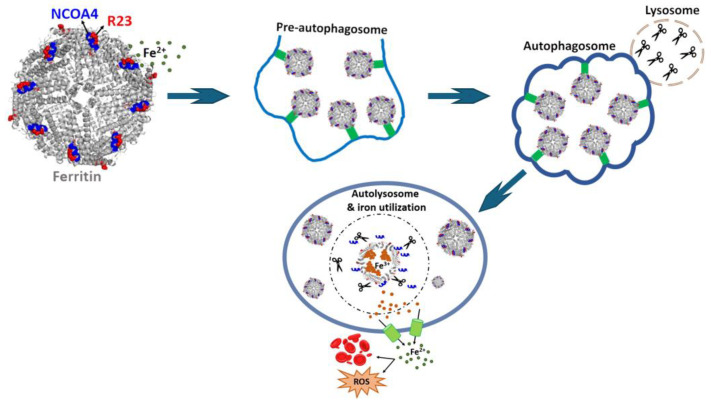
Ferritin–NCOA4 complex and NCOA4-mediated ferritinophagy. For clarity, only the NCOA4 fragment (383–522) that specifically binds to the exposed Arg23 residue of the H subunit of ferritin is shown. The scissors symbolize the lysosomal protein degradation process.

The reverse process—how iron stored in ferritin is made available to cells—was an ancient problem, originally raised by Granick, that presented a significant challenge. Direct solubilization of the iron core was thought to involve reduction reactions, and a ferritin reductase was described in early research by Zaman and Verwilghen [150] but never confirmed. Since the released divalent iron could produce harmful ROS, releasing iron from ferritin in a safe manner is crucial for cellular health. Early studies suggested that ferritin is degraded in the lysosome to release iron, with a mechanism differing in iron-depleted and iron-replete cells [151]. However, the breakthrough came with the discovery of ferritinophagy by Mancias et al., a specific autophagic process. In this pathway (Figure 4), the protein NCOA4 binds the H chain of ferritin and transports it to lysosomes, where ferritin is degraded and the stored iron is solubilized and recycled back into the cytosol [152]. This iron-regulated mechanism (Figure 2) offered a clearer understanding of how ferritin participates in iron recycling and contributes to cellular iron homeostasis. Interestingly, similar mechanisms seem to be involved in ferritin export. Macrophages, which were identified as the primary source of extracellular ferritin [153], secrete ferritin through a NCOA4-independent lysosomal pathway involving secretory autophagosomes [154] as well as via the multivesicular body–exosome pathway [155].

### 5.5. Discovery of Ferroptosis

Ferritin’s role in cellular protection includes apoptosis, where it has been shown to inhibit cell death by suppressing ROS production, additional evidence of the antioxidant role of ferritin [156]. However, a more critical connection between ferritin and programmed cell death emerged with the description of ferroptosis in 2012 [157]. Ferroptosis is a distinct form of programmed cell death caused by lipid peroxidation, facilitated by iron. This discovery has sparked considerable interest, as ferroptosis has been linked to various diseases, including cancer and neurodegeneration [158]. In this context, the iron sequestering capacity of ferritin might be key in regulating ferroptosis, as shown by the ferroptosis-mediated cardiomyopathy triggered by H-ferritin gene deletion [159]. The role of iron and iron-related proteins, including ferritin, in regulating this pathway has generated extensive research into the mechanisms of ferroptosis while also driving efforts to identify agents that can either promote or inhibit this process. The uncovering of ferritinophagy and ferroptosis has expanded the understanding of ferritin’s biological roles, showing that it is not only an iron storage molecule but also a dynamic regulator of cellular iron homeostasis and a key player in cell survival and death mechanisms.

### 5.6. Other Complex Ferritin Functions

Studies on conditional H-ferritin KO mice, produced by Lukas Kuhn’s laboratory, revealed novel tissue-specific roles of ferritin. In 2010, these authors showed that intestinal H-ferritin is necessary for regulating iron absorption, aligning with the “mucosal block” theory [160]. Additionally, deleting H-ferritin in mouse bone marrow was shown to reduce B- and T-lymphocyte populations, supporting its role in immune function [161]. Interestingly, KO mice for L-ferritin exhibited no significant phenotype, consistent with clinical data showing that mutations affecting L-ferritin’s start codon produce no hematological or neurological symptoms [162].

Ferritin’s ferroxidase activity and its role in iron storage are involved in a wide range of biological processes (Table 1). In human and animal models, H-ferritin plays an inhibitory role in osteogenesis [163] and is involved in a chemokine receptor CXCR4-dependent dysfunction in neurons due to opiates [164]. Furthermore, it stabilizes hypoxia-inducible factor-1α (HIF1α) under lipopolysaccharide activation in oxygen-rich environments [118], showcasing its regulatory role in cellular responses to inflammation and oxidative stress. Moreover, upon endocytosis, ferritin-H subunits stimulate the formation of the NLRP3 inflammasomes in liver stellate cells to drive hepatic inflammation [120].

Studies of ferritin across different species revealed some surprising findings. In marine diatoms, for instance, ferritin plays a crucial role in providing iron, which contributes to their ability to form blooms [165]. In bay scallops, ferritin is involved in immune defense mechanisms, while in marine phytoplankton, it regulates iron homeostasis [166]. Interestingly, ferritin has also been linked to light production in the marine worm *haetopterus* [167], and to the regulation of mitophagy in oysters [168], highlighting its diverse roles across different marine species.

### 5.7. Recent Biotechnological and Clinical Applications

The unique structural properties of ferritin have been leveraged in various innovative approaches and for different applications. Cohen et al. [169] demonstrated that ferritin overexpression in cells could be monitored using magnetic resonance imaging (MRI), as the accumulation of iron lowers T1 and T2 signals, showcasing the utility of the iron core’s magnetic properties for tissue identification. This characteristic has allowed ferritin to be used as an endogenous MRI reporter for the noninvasive imaging of gene expression in several studies. One notable example is the use of MRI to quantify signals in transgenic grafts overexpressing ferritin in murine myocardial infarcts [170]. Beyond imaging, the magnetic- and temperature-sensitive properties of ferritin’s iron core have been exploited in more advanced applications, such as the magneto-genetic manipulation of proteins and organelles within living cells, facilitated by engineered ferritin [171]. On the technological front, ferritin nanoparticles have been incorporated into organic field-effect transistor memory devices [172]. Moreover, ferritin remains a model system for protein crystallization studies and advancements in cryoelectron microscopy (Cryo-EM) [173].

**Figure 5:**
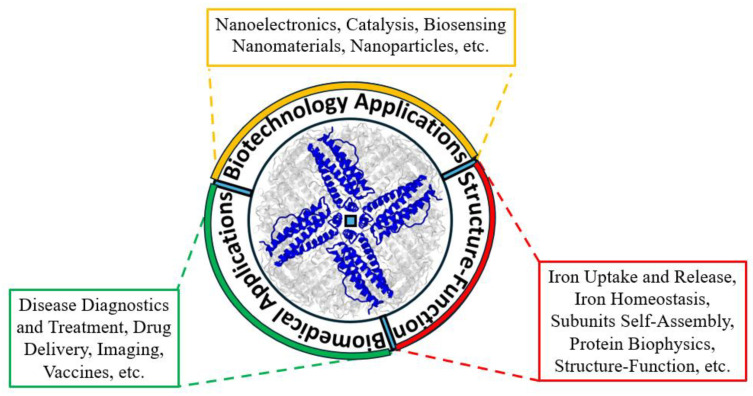
A scheme summarizing the different types of functions and applications of ferritin. In addition to its physiological role in controlling cellular iron homeostasis and ferroptosis, the protein has found application as a biosensor, in nanoelectronics, in theranostics for drug delivery, imaging, and vaccine development.

Furthermore, the ferritin nanocage structure possesses remarkable properties, including high stability and the ability to encapsulate drugs or chemical agents within its cavity. Such properties are currently being exploited in the medical field for theranostic applications (Figure 5). As a drug delivery system, drug-loaded ferritin nanoparticles have shown higher efficacy in targeting cancer cells compared to free drugs [174], especially since TfR1, which binds H-ferritin, is highly expressed on the surface of many cancer cells. Additionally, ferritin is being explored as a platform for vaccine development, with epitopes genetically fused or chemically attached to ferritin molecules to boost immunogenicity [175]. Ferritin’s clinical relevance surged during the COVID-19 pandemic, as serum ferritin levels became a key prognostic marker of disease severity [176], reflecting its central role in inflammatory and other iron-related pathways. Overall, ferritin’s unique properties have made it an indispensable tool across various scientific disciplines from bioengineering and drug delivery to immune research and materials science, as shown in various recent reviews [177,178,179].

## 6. Conclusions

Over the past 80 years, with more than 40,000 original papers and hundreds of review articles, our understanding of the ferritin molecule has advanced significantly, as indicated by the milestones summarized in Table 2. Ferritin is present in nearly all living organisms, likely since the early stages of life, underscoring its evolutionary significance. Its fundamental structure, a highly conserved alpha-helix bundle forming a spherical cage, has remained unchanged, despite major sequence variations across different species. This structure has enabled ferritin to take on various specialized roles depending on the organism and cellular context.

This short history of ferritin shows how its research has developed, starting before World War II, and then progressing constantly with the development of new techniques. It also reveals that the interest in ferritin has never declined; on the contrary, it kept increasing over time in parallel with research on iron homeostasis, in which ferritin has a key role. This field is becoming increasingly important since iron is involved in various disorders, including neurodegenerative diseases, cancers, and inflammation. Ferritin is also attractive for various other reasons, including its use as a tracer in EM or NMR, the unique properties of its iron core, its potential for drug delivery, its ability to carry epitopes, and its capacity to interact with cells via specific receptors.

The current interest in ferritin is primarily focused on its role in ferroptosis and on its various applications in biotechnology and nanomedicine. However, several aspects remain poorly understood, such as its extracellular functions and the origins and specific functions of serum ferritin, despite ongoing research. We hope that this reflection on the history of ferritin will inspire further studies on this fascinating molecule.

## Figures and Tables

**Table 2:** Milestones in ferritin research.

Year	Milestone	Reference
1937	Ferritin crystallization	Laufberger [5]
1942	Apoferritin	Michaelis [6]
1948	Vasodepressive material	Mazur-Shorr [11]
1951	Ferritin iron storage	Granick [7]
1954	Electron microscopy	Farrant et al. [15]
1971	Isoferritins	Urushizaki et al. [47]
1972	Serum ferritin	Addison et al. [52]
1978	H and L subunits	Arosio et al. [50]
1983	Monoclonal antibodies	Cavanna et al. [63]
1983	Cloning cDNAs of ferritin subunits	Brown et al. [68]
1984	Solving crystallographic structure of ferritin	Ford et al. [65]
1987	Production of recombinant ferritin	Levi et al. [74]
1987	IRE identification	Hentze et al. [81]
1988	IRP purification	Walden et al. [82]
1988	Ferroxidase activity in H chain	Levi et al. [76]
1990	Bacterial heme ferritin BFR	Kadir & Moore [104]
1991	Identification of ferroxidase center	Lawson et al. [78]
1997	Dodecameric DPS	Bozzi et al. [105]
2000	H-ferritin KO mice	Ferreira et al. [134]
2001	Neuroferritinopathy	Curtis et al. [135]
2001	Mitochondrial ferritin	Levi et al. [136]
2008	Iron chaperone to ferritin	Shi et al. [149]
2012	Ferroptosis	Dixon et al. [157]
2014	Ferritinophagy	Mancias et al. [152]
2021	Recombinant human isoferritins	Srivastava et al. [148]
2024	Structure of isoferritins	Bou-Abdallah et al. [66].
